# Datafication and accountability in public health: Introduction to a special issue

**DOI:** 10.1177/0306312719860202

**Published:** 2019-08-05

**Authors:** Klaus Hoeyer, Susanne Bauer, Martyn Pickersgill

**Affiliations:** Centre for Medical Science and Technology Studies, Department of Public Health, University of Copenhagen, Copenhagen, Denmark; Centre for Technology, Innovation and Culture, University of Oslo, Oslo, Norway; Centre for Biomedicine, Self and Society, University of Edinburgh, Edinburgh, UK

**Keywords:** accountability, big data, data, datafication, population, public health

## Abstract

In recent years and across many nations, public health has become subject to forms of governance that are said to be aimed at establishing accountability. In this introduction to a special issue, *From Person to Population and Back: Exploring Accountability in Public Health*, we suggest opening up accountability assemblages by asking a series of ostensibly simple questions that inevitably yield complicated answers: What is counted? What counts? And to whom, how and why does it count? Addressing such questions involves staying attentive to the technologies and infrastructures through which data come into being and are made available for multiple political agendas. Through a discussion of public health, accountability and datafication we present three key themes that unite the various papers as well as illustrate their diversity.

## An STS of, and for, public health

In recent years and across many nations, public health has become subject to forms of governance said to be aimed at establishing accountability. It has given rise to what Hogle (2019) in her contribution to this volume regards as *assemblages* (i.e. ‘arrangements of practices, technologies, and theories that configure action in a sociotechnical space’) of accountability. Important ideas about populations and groups emerge through such ‘accountability assemblages’, and they shape the rights and duties of the individuals who come to be defined as belonging to such groups (Taylor, 2017; Taylor et al., 2017). The involved profiling practices are technology intensive and politically volatile. The data infrastructures and data practices emerging with reference to accountability often provide the basis for knowledge claims about needs, causes, and effects relating to health and illness. In some instances, population data also serve to hold powerful actors responsible for their use and prioritizations of resources. Such data practices are said to create accountability, but what does that word mean, and what does it do? How do these practices affect prevention, healthcare and the state of public health?

This special issue explores accountability in public health from the perspective of Science and Technology Studies (STS). We believe that it is time to gather STS work that makes visible the diverse dynamics of accountability assemblages and therefore present case studies of the data politics of accountability involved in public health and the population health sciences. The contributions grew out of a panel originally convened for the 4S/EASST Annual Meeting held in Barcelona in 2016. The discussions during the meetings illustrated how STS has a lot to say about public health and accountability – as well as the processes of ‘datafication’ (Ruckenstein and Schüll, 2017) through which the two unite. Science and technology mediate accountability assemblages at all levels: One cannot understand the workings of accountability discourses without engaging the material practices through which they emerge and come to have effects. By engaging each other’s work, we learned that ostensibly straightforward empirical questions can open up intricate issues: What is counted? What counts? And to whom, how and why does it count? The issues revealed affect the livelihood and well-being of millions of people, enjoining renewed reflections on classical topics in STS.

By bringing together papers that all relate to public health, accountability and processes of datafication, we illustrate a diversity of practices in different public health settings in wealthy countries situated in what is also sometimes referred to as ‘the Global North’. We briefly highlight the topic of each paper (see the respective abstracts for a more elaborate account), and then elaborate on themes uniting them.

In their contribution to this volume, Amelang and Bauer (2019) present a study of a cardiovascular risk score and its translation to preventive medicine in Germany. They analyze how epidemiological risk scores work as ‘accountability devices’ and show how they, despite their algorithmic character, continue to be in the making. Cool (2019) explores what she terms ‘accountability anxiety’ as a particular reaction among population researchers in Sweden to European data regulation and potentially conflicting demands of data protection and data sharing – thus showing how researchers negotiate the burden of compliance, legal uncertainties, and ethical concerns. Hoeyer (2019) discusses the politics of personalized medicine in the hyper-digitalized Danish healthcare system and suggests that data might be useful not so much for the information they convey, but for the way in which they deliver a promise of future knowledge, one that serves to postpone calls for present action on inequality in health. Hogle (2019) outlines the work of Accountable Care Organizations in the US that are expected to deliver ‘value-based care’ and new forms of accountability, while simultaneously creating potentially enduring impacts by facilitating data infrastructures for individual profiling. Kalender and Holmberg (2019) analyse the everyday work going into the making of population data by following a German cohort study and suggest that the big numbers of epidemiology in a sense also depend on small talk. They show how it takes a lot of ‘courtesy work’ to keep participants enrolled. In their article here, Kerr et al. (2019) describe the development of polygenic risk stratified cancer screening in Europe, North America, and Australia, and argue that the emerging population tools create new stratifications and forms of responsibility. Finally, Pickersgill (2019) explores the proliferation of psychological therapy in recent years in the UK as a consequence of strategic uses of population data and accountability. He suggests that this shift can, to an extent, be regarded as an example of de-biomedicalization. Big numbers can sometimes give room for therapeutic talk.

We begin the introduction with a discussion of the term ‘public health’ and how it is emerging as a topic in STS. Then we examine the notion of accountability and its relation to classic issues about power and knowledge, and take this as a stepping stone to a discussion of the role of metrics and datafication as they currently play out in this field.

## What is public health and why is it important for STS?

Public health is a term that has acquired three related, but still distinct meanings. First, it is used to denote a state of health and illness in a population. Second, public health is a scientific field aimed at ascertaining that state, identifying its determinants and understanding the implications of attempts to modify it. Determinants have typically been much more broadly conceived than in laboratory science or clinical medicine. They include sanitation, food security, environmental factors, and social, economic and political factors. This meaning of ‘public health’ can also be referred to as ‘public health science’ or ‘population (health) science’, with ‘science’ sometimes self-consciously pluralized as ‘sciences’ in order to underscore the heterogeneity of epistemologies and knowledge practices comprising this epistemic complex (e.g. medical sociology, molecular epidemiology, health economics, and so on). Third, public health can be taken to mean political programs and infrastructures (existing or lacking) aimed at improving the state of health in a population (however that population is constructed). Scholars sometimes slide unnoticeably between the different meanings and move from insights into inequalities in health and illness and their related determinants to arguments of political necessity. The slippage between knowledge, activism and political intervention signals the enduring presence of the classic biopolitical tension between power and knowledge characteristic of the wider field of public health; it is a well-established insight that populations are counted to be governable (Asad, 1994; Bowker and Star, 1999; Deleuze, 1992; Desrosiéres, 1998; Sætnan et al., 2011).

### The techno-politics of population knowledge

In population health science, the object of knowledge is not the individual, but a pattern or a rule (Armstrong, 2017). The populations dealt with are products of particular technologies of counting and modeling (Bauer, 2013): different populations emerge from different modes and technologies of counting (Grommé and Ruppert, 2019; Hacking, 1982; Ruppert et al., 2017). These technologies involve definitions and operate through materially embedded forms of creating data as well as curating, storing, distributing, and analyzing them. How data is produced and what is done with it are shaped also by political and economic contexts. For example, in the Nordic countries, the socio-material and economic conditions facilitated the establishment of national ‘registries’ (Bauer, 2014; Reinholdt, 2018), while population-level knowledge in Malawi came to involve very different material practices dependent on paper-based surveys, fieldworkers and global economic funding schemes (Biruk, 2018). When the Gates Foundation funds the establishment of data infrastructures throughout much of Africa (Adams, 2016a; Erikson, 2016), they are construed in manners that differ significantly from data infrastructures in China or the US (Creemers, 2016; Deibert and Pauly, 2019). How public health law, policy, and practice operate is profoundly shaped by the social and material infrastructures through which data on health needs are generated and populations come into being as objects of knowledge. This special issue presents examples from infrastructures and practices enacted: in the US (Hogle, 2019), the UK (Pickersgill, 2019), Sweden (Cool, 2019), Germany (Amelang and Bauer, 2019; Kalender and Holmberg, 2019), Denmark (Hoeyer, 2019), and through international, partly commercial, networks (Kerr et al., 2019). We illustrate the diversity of practices in different public health settings to illustrate how STS can investigate technologically mediated construction of populations and the translation of this knowledge into societal action.

During the past two decades, a molecular turn in the population health sciences has created extensive infrastructures of biobanks through which another set of ideas about both population and individual emerge (Ackerman et al., 2016; Hoeyer, 2002; Radin, 2017; Tupasela et al., 2015; Tutton, 2004). This volume illustrates how infrastructures for population-based genetics can also emerge out of developments in clinical care or commercial rearrangements. Such infrastructures shape research options and thereby the patterns – and the ‘populations’ – that can be ‘discovered’. Another significant trend in public health has been the transition from focusing on mortality (the statistics of life and death) to morbidity (the relative burden of disease and quality of life measurements) (Moreira, 2019; Wahlberg and Rose, 2015). This focus on disease burden and life-long risk is co-produced with tools of measurement and identification of those at-risk and in need of additional treatment (Amelang and Bauer; Kalender and Holmberg; Hogle, this volume). By defining and investigating public health in particular ways, political options are shaped and particular futures made possible.

STS has contributed greatly to understandings of processes of numeration (Martin and Lynch, 2009; Porter, 1995), and the performative work of algorithms, digitization and big data (Denis and Goëta, 2017; Edwards et al., 2011; Jaton, 2017; Stark, 2018). All of these developments are central for understanding the field of public health, and yet public health research and practices have not been subject to the same close scrutiny as traditionally more dominant modes of technoscience and medicine – laboratory research, for instance (Latour and Woolgar, 1979). If laboratory science and engineering often work with translations of models from bench or desk into practice and deliver products for purchase and consumption, the field of public health often depends on non-research infrastructures for data collection, and promotes results through policy changes, rather than as sellable goods – although data-driven innovation in public health is today often enjoined precisely as a means of bringing the population health sciences into alignment with markets. It reverses a number of the dynamics that have been studied in STS.

In this volume we describe different types of technological mediation of data practices from seemingly simple checklists (Pickersgill, 2019) and documentary tools (Cool, 2019), to risk scoring (Amelang and Bauer, 2019; Hoeyer, 2019) and sophisticated algorithms that remain black-boxed to many of their users (Hogle, 2019; Kerr et al., 2019). These mediations are techno-metricized accountabilities. They are informed by politics, yet they cannot be captured and described with reference to political intention alone. They operate on and through both human and nonhuman actors, and participate in, inflect, and sometimes steer wider socio-political action (Erikainen et al., 2019; Petersen, 2019; Watcher, 2017). Relevant to all of them are the symbolic and material processes through which public health research and policy are represented and understood as legitimate and authoritative – how public health is made accountable. Who is held accountable, through which means, and for what?

## Accountability: The intersection of knowledge and politics

Accountability is a matter of knowing, doing and becoming. In public health, accountability assemblages are archetypical instances of biopolitics, defining who needs to know – and do – what, and for and to whom (Foucault, 1991; Lemke, 2007; Rose, 2007). When, in the early 20^th^ century, Max Weber (1947) described the accountable public servant, he emphasized the need for secrecy – an *absence* of data sharing (p. 233). A responsible bureaucrat needed to document his (sic!) practice, but he also needed to be guarded against volatile political interference. The closed archive was emblematic of this dual ambition. If, at the time of Weber, the meaning of accountability centered on the private room for judgment handled by public professionals, then by the end of the 20^th^ century it had become associated with system-level relations and obligations depending on *access* to data documenting the public service delivery (Bessette, 2001). Today, in many (perhaps even most) nations, data collection and data sharing are more or less taken for granted in political discourses on accountability in healthcare. The closed archives have been replaced by data infrastructures and varying forms of digital surveillance (Moore, 2018). Twenty-first century discourses of accountability propagate rhetorics of ‘open government’ (The White House, 2013) and ‘transparency through digitalization’ (Europa-Kommisionen, 2017). In healthcare, the emphasis is on ‘learning health-care systems’, using digital data to enhance and monitor performance (Olsen et al., 2007). None of these discourses would have any resonance, however, without technologically mediated data infrastructures promising to make data available for real-time surveillance (Goëta and Davies, 2016). Technologies are the foundations on which organizations may promise to produce ‘answers’ – if not now, then in an undefined future (Hoeyer, this volume).

### The multiple meanings of accountability

The tension between individual and system-level responsibility also can be identified at the level of official definitions. Etymologically, accountability can be traced to medieval theological discussions. In the 18^th^ century, accountability entered secular parlance as a ‘state of being answerable’ (*Online Etymological Dictionary*). To be ‘answerable’ conveys an obligation to speak and to let oneself be investigated, and in this sense accountability clearly refers to relations. Furthermore, account-ability denotes both the sense of a moral obligation to answer and a practical ability to deliver that answer. An answer might demand extensive work to produce the needed knowledge. Relations of accountability thus involve moral relations that can instigate documentary and analytical work dependent on science and technology.

The *Oxford English Dictionary* defines accountability with reference to an individual, but then expands it to an aggregate level:

The quality of being accountable; liability to account for and answer for one’s conduct, performance of duties, etc. (in modern use often with regard to parliamentary, corporate, or financial liability to the public, shareholders, etc.); responsibility.

According to the *Cambridge Dictionary*, accountability defines ‘a situation in which someone is responsible for things that happen and can give a satisfactory reason for them’.

In other European languages, we similarly find moral valances. One common German translation is Rechenschaftspflicht, which has a legal and moral tone to it emphasizing legal obligation and duty. The respective French terms closest to accountability are ‘responsabilité’ (most frequent translation) and the phrase ‘rendre des comptes’, with responsabilité pointing to institutional responsibility and liability after damage, as well as moral and political responsibility, while the (more literally) related terms ‘compter’ is about to matter and to count, whereas ‘comptabilité’ is about financial bookkeeping (resonating also with the English accounting and the German Rechenschaft). The Danish translation is ‘ansvarlighed’ (meaning responsibility-ness, or the state of being responsible), but the English word ‘accountability’ is often used in Danish policy texts or prose about governance. Similarly, the Swedish word is ‘ansvarig’ (see also Cool, 2019), and the Norwegian is ‘ansvarlighet’, all of the same roots, and all with a focus on responsibility.

Though accountability denotes responsibility across a number of languages, it of course remains negotiable who are responsible to whom and for what and how this is being emplaced (Jerak-Zuiderent, 2015; see also the contributions to this volume by Cool, Hoeyer, Hogle, and Pickersgill). Nevertheless, it is safe to say that accountability is a concept that intervenes in relations and in politics, as much as it describes these relations or political situations. In the area of health, it comes to mediate relations of care. As Pols (2006) notes, ‘different styles of accounting evaluate and legitimize care while structuring notions of what good care is’ (p. 409). The concept is politically expedient partly because of its ability to communicate a moral sense of obligation: by referring to accountability, political actors can act on, shape or change relationships.

### Debates about accountability in public health

Obviously, the theme of accountability features prominently also in debates about public health (in all three meanings) (Beitsch and Corso, 2009; Kraak et al., 2014; Wholey et al., 2010). One theme in public health debate is the issue of *purpose* in allocating a responsibility (Fahlquist, 2006): Should it be to ensure good outcomes (e.g. through incentives for prevention), or to ensure fair attribution of blame (e.g. by holding those believed to be the cause of illness responsible)? Examples of this conflict are discussed in several of the contributions to this volume (e.g. in the articles by Hoeyer, Hogle and Kerr et al.).

Another theme in the population health science literature on accountability revolves around *who* should set the goals of the accountability assemblages (Merry, 2016). For instance, the US market-based system emphasizes economic accountability in negotiations between third-party payers and service deliverers (Hogle, 2019), while state-centered European systems tend to focus on standardization of care (Ashmore et al., 1989). In global health, donor accountability and distribution of responsibility among funders and local partners have been intensely debated (Adams, 2016a), but in their article in this volume Kerr et al. (2019) also point to global commercial assemblages that diversify responsibilities in new ways.

Yet another theme is *for what* actors should be held accountable: e.g. patient-centered outcomes and quality standards, a wider sense of community health, human rights, or levels of participation (Van Belle and Mayhew, 2016)? In this volume, Cool shows how European data regulations make researchers contemplate towards whom they are accountable, while nobody is really accountable for helping the researchers with meeting the new obligations. Hogle illustrates tensions between being responsible for fiscal balance and good care, and Pickersgill shows how feeling accountable for delivery of needed care can involve gaming accountability assemblages to appear ‘effective’ according to criteria one might not otherwise subscribe to or agree with.

Finally, an important theme has emerged in relation to *how* accountability questions can be asked and what may count as epistemic bases for claims made. Controversies about childhood vaccination have given such epistemic controversies a very public form and simultaneously provided vivid examples of the messy relationships between states, publics and citizens, as well as between individual liberty and personal accountability, that play out when the public health of entire countries is at stake (Decoteau and Underman, 2015). In this volume, Amelang and Bauer as well as Kalender and Holmberg, explore the making and translation of epistemic tools connecting individuals to population averages and population data to individuals. Taken together, these contributions illustrate the intricate dynamics of accountability assemblages, and the need to stay attuned to the specificities of each case. Accountability can be a double-edged sword.

### The audit explosion: Economic, legal and numerical responsibility

The Google database of digitized English language books has a function, Ngram, through which a graph can be generated indicating the prevalence of words in selected time periods. An Ngram of the appearance of the word accountability (included in the Ngram database) indicates that modest but regular usage began in the early 19^th^ century. The term was then used steadily until around 1970, when it suddenly became very popular and from the 1980s it grew exponentially in use. The sudden exponential growth might reflect the deployment of the term in programs of modernization of the public sector typically associated with the vague term ‘neoliberalism’ and what Strathern (2000) has called the emergence of an audit culture and Power (1997) an audit society. Auditing, again, depends on and propagates investments in the creation of documentary technologies that accumulate and preserve data for economic, organizational and legal purposes (Bevan and Hood, 2006; Wadmann et al., 2013). Through its association with audits, accountability came to focus heavily on financial and legal accountability. The prominence of metrics in discourse about accountability emerged along with the growing popularity of the financial meaning of the term from the 1980s onward (Erikson, 2012; Sangaramoorthy and Benton, 2012; Storeng and Behage, 2017; Sullivan, 2017; Tichenor, 2016). It has implied an intensified datafication of public health, and it is to the metrical practices in public health (in all three meanings of the term) we now turn.

## The datafication of public health

If accountability has come to be related to counting, what does it mean to count? Martin and Lynch (2009) note that ‘counting’ initially appears to be a mundane and straightforward activity, yet it demands intricate systems of classification to count and such systems can be highly context-dependent and ambiguous. The world does not come in readymade packages that can be counted; to count in a domain, it must be possible to delineate the domain’s objects, and the agents counting must have access to relevant data. The matching of categories to the amorphous world has been a foundational problem for philosophical epistemology, as famously pointed out by Foucault (1999) in *The Order of Things*. Kant (2017 [1781]), for one, struggled to establish foundational grounds for objects of knowledge, while dismissing a purely empiricist argument; his solution was to be found in a priori structures of reason that should determine what could be meaningfully made into an object of knowledge.

Whereas Kant sought a priori structures for reason, Foucault focuses on the historical contingency of the involved forms of reasoning. Work on the history of statistics and population knowledge has followed the Foucauldian line of inquiry, exploring how political institutions for the production of population knowledge have shaped the ‘objects’ that are counted (Desrosiéres, 1998, 2011; Hacking, 1990; Plant, 1998; Porter, 1995). This work has shown how it is a political as much as an epistemological achievement to establish a statistical object (Moreira, 2019). Populations do not simply exist as objects of knowledge, but are products of data practices (Moreira and Palladino, 2011). In Desrosiéres’ (1998) words, ‘The aim of statistical work is to make a priori separate things hold together, this lending reality and consistency to larger, more complex objects’ (p. 236). Statistics work from data, but these first need to be produced (Biruk, 2018; Gitelman and Jackson, 2013). Data are not raw materials; they are, as suggested by Latour (1999), achievements (p. 42). This special issue contributes to the study of these achievements and thereby to the opening up what is all too often described as a simple process of ‘datafication’, as when big data proponents state: ‘To datafy a phenomenon is to put it into a quantified format so it can be tabulated and analyzed’ (Mayer-Schönberger and Cukier, 2013: 78). We do not dismiss or criticize the usefulness of datafication per se (Muller, 2018), but we emphasize the need to understand better when it is valuable, for whom, and for which purposes. In some instances, new data infrastructures can create visibility (Ginsburg and Rapp, 2017; Rabeharisoa et al., 2014), and in others they involve forms of decontextualization of information in ways that facilitate deliberate or unintended unknowing (Geissler, 2013) or ignorance (McGoey, 2012).

### From epidemiology to big data

Epidemiology delivered the core method of population health science and epidemiologists drew on big data sets long before big data came into fashion (Bauer, 2014; Hoeyer, 2019). The emergence of clinical epidemiology was foundational for the creation of the movement called evidence-based medicine (Lambert, 2006), yet the basic extrapolation from the population level to individual patients or citizens continues to be contested (Armstrong, 2017; Holmberg et al., 2013). Epidemiologists have pointed out how population data cannot predict individual outcomes, and some epidemiologists talk about the replacement of statistical probability with individual prediction as the ‘ecological fallacy’ (Henderson and Keiding, 2005; Piantadosi et al., 1988). The troubled link between individual and population also means that public health scholars acknowledge that it is often necessary to treat a given number of persons who will not directly benefit as individuals, to reach population-level effects (Brodersen et al., 2018). People are treated or expected to engage prevention in pursuit of an effect that can be detected at the population level, but not necessarily by each and every individual. Sociologists have criticized how, as a consequence, individuals are held responsible for the achievement of population goals (Armstrong, 2017; Lupton, 1997). Several articles (see contributions by Amelang and Bauer, Hoeyer, and Hogle) in this volume present examples of individualization of responsibility, and, in particular, Kerr et al. show that individualization of responsibility can take many forms; techno-political accountability assemblages can diversify, expand and stratify responsibility in new ways.

The so-called big data movement has given the ecologic fallacy renewed traction. Profiles based on aggregate data from multiple domains (including data previously unrelated to health), are increasingly used in individual-level practices (Hoeyer, and Hogle in this volume). In the big data movement, former distinctions between the population level and the individual level collapse and correlations are made to work as tools of predictions irrespective of the expectations in classic epidemiology of models, theory and controls (Stark, 2018). Lury and Day (2019) suggest that it has created an ‘age of personalization’, where population data compile ‘types’ and use the unique patterns of such characteristics to rank and market individuals and organizations. Such data-intensive profiling tools are marketed as key to the delivery of ‘public health management’ (Hogle, this volume). They also mark a break with a 400 year monopoly of the state on population data and a transition to a more fluid and market-based form of knowledge production in the population sciences (Bigo et al., 2019, Kerr et al., 2019). Zuboff (2019) even suggests that they form part of a new mode of production – surveillance capitalism. Our point here is more limited and relates to the politically ambiguous effects of new data assemblages: Individuals at risk and in need of help are simultaneously framed as a risk to the population – with respect to the aggregate state of health and as financial risk to the healthcare system and the payers (see Hoeyer, and Hogle, in this volume). This revivifies the possibilities of punitive effects on individuals of the kind that public health law, policy and practice have always wrestled with.

### Global ramifications

The datafication of public health is a phenomenon with implications far beyond the so-called Global North. In 2015, the United Nations (UN) General Assembly decided on 17 Sustainable Development Goals (SDGs), broken down into 232 indicators, to define medium-term directions for international collaboration on health, poverty, gender equality and environmental sustainability. The datafying logic and format of SDGs can also be found in another very influential UN report, *A World That Counts* (The United Nations Secretary-General’s Independent Expert Advisory Group on a Data Revolution for Sustainable Development, 2014). It states that ‘[i]mproving data is a development agenda in its own right’ (p. 3), and explained that this is because ‘[d]ata are the lifeblood of decision-making and the raw material for accountability’ (s. 2). The report also proclaimed an ostensibly new vision for global public health: ‘Never again should it be possible to say “we didn’t know”. No one should be invisible. This is the world we want – a world that counts’ (p. 3). In this way, the logic of datafication as a tool for accountability is here taken to an extreme with the belief that it is a special task for metrics to make human suffering visible and that it is through metrics only that the international community can be held accountable. From this perspective, to be seen, known and acknowledged *is* to be counted.

Despite the breathlessness of the UN report, and its presentation of data-driven public health as novel and vital, the notion that global organizations need to set goals and measure them has a significant history. Halfon (2006) has argued that one key element in this process of datafication (Ruckenstein and Schüll, 2017) was the 1994 report of the *International Conference on Population and Development* (also known as the Cairo consensus document). It set goals and standards for measuring whether they were achieved in relation to population developments, reproductive health and family planning. According to Murphy (2017), in this period ‘population’ and ‘development’ became political technologies in their own right, contributing to a larger process of economizing life. At the time, its proponents called it the ‘largest social science research project ever undertaken’ (in Halfon, 2006: 792). Standards were developed and implemented across the globe to stabilize measurable phenomena and make them governable. Halfon nevertheless argues that, despite calling it a ‘consensus document’, consensus did not mean agreement. Rather, the report operated more like ‘a metaphor for a particularly robust network, one that allows various actors to act “as if” they were all doing and thinking the same thing (that is, “as if” there were unity)’ (p. 784). The report achieved no global consensus, but a new way of overcoming the vivid and fierce disagreement between different countries (and subgroups within them, not least in the US). New technologies of large-scale data collection emerged through these initiatives and they have come to play a central role in contemporary ideas about accountability at both global and national levels of government.

While Isin and Ruppert (2019) argue that there might have emerged a distinct data politics of the Global South, we point to continuities as well as differences both between and within the Global South and the Global North. We thereby highlight the importance of understanding the pervasiveness of accountability assemblages in public health globally as well as the need to appreciate the situatedness and distinct implications of each socio-material setup to create ‘accountability in public health’. There is a continued need for STS work, to hold accountability assemblages accountable irrespective of where they emerge.

### Beyond numbers

The popularity of big data and the datafication of global public health can result in an elision of analytic attention to how accountability assemblages work through *discursive* as well as numerical logics. Data shape but do not determine political developments (Wyatt, 2008). Numbers tend to be used selectively and, as Adams (2016a) remarks, ‘number crunching and metrics work as, in their own way, forms of storytelling’ (p. 9). Hoeyer thus describes a public health accountability assemblage legitimized with reference to the need for numbers and ‘knowledge about what works’, but that invents the numbers it uses to justify the suggested policies and disregards existing evidence on preventive methods. Cool describes how the so-called ‘accountability principle’ in European data regulation stimulates reflections and narrative accounts of responsibility among researchers trying to decide who they are accountable to and in which way. Data here produces words, as much as the other way around. Kalender and Holmberg describe the courtesy work needed when researchers interact with participants in cohort studies and thereby illustrate how even large numbers emerge through words (see also Ackerman et al., 2016). A similar point could be made about coding and classification in most areas of clinical healthcare where health professionals must know how to engage patients to get the information needed for a diagnosis (Jutel, 2009). Pickersgill provides a telling example of the way in which numerical forms of evidence have been used strategically to install and legitimize psychological therapy as standard treatment where pharmaceutical products used to be the default choice. Here numbers have created a space for words in a very literal sense.

If accountability is a relationship in which someone can be asked to deliver an account for something, this can – in principle at least – always take the form of a narrative and not just numbers (Brenneis, 2006; Riles, 2006), and taken together these papers illustrate how the implications of datafication are unpredictable and depend upon much more than what numbers contain and convey.

## Coda

Without technologies for data collection and computation, population facts do not exist (O’Riordan, 2017). It is through data-saturated and technology-mediated practices that populations are performed (cf. Law, 2009; Ruppert, 2012). Public health is accordingly saturated with computational technologies and calculative devices mobilizing and performing accountability metrics (Callon and Muniesa, 2005). Without ‘population facts’, there are no objects of accountability for ‘public health’ (here in the meaning of as a state of well-being in a population). How population facts are produced and used to produce different versions of accountability therefore deserve continued STS attention. There is not one single process of ‘datafication’ in accountability assemblages, but different systems generating different objects of concern through different ways of counting and by holding different actors accountable. These assemblages continue to work through both words and numbers and they are always materially mediated.

With this volume we therefore hope to stimulate even more empirical studies of the data politics of accountability in public health. Accountability assemblages serve to establish responsibilities. Accountability typically ties together group and individual through a circular movement from the individual to the population and back (Holmberg et al., 2013). This movement depends on technologies of documentation and computation. It can be opened up and studied. Depending on differing ideas about who should be held responsible for what – and by whom – different accountability assemblages emerge along with very different data infrastructures. This special issue presents some of these differences. It is meant to orient STS towards public health as a central concern for the discipline, and to show how there is a special role for STS in holding accountability assemblages accountable.

## References

[bibr1-0306312719860202] Ackerman SL Darling KW Lee SS-J, et al (2016) Accounting for complexity: Gene–environment interaction research and the moral economy of quantification. Science, Technology, & Human Values 41(2): 194–218. 10.1177/0162243915595462 PMC8388243 34456398

[bibr2-0306312719860202] Adams V (2016a) Introduction. In: Adams V (ed.) Metrics: What Counts in Global Health. Durham, NC: Duke University Press, 1–17.

[bibr3-0306312719860202] Adams V (2016b) Metrics of the global sovereign: Numbers and stories in global health. In: Adams V (ed.) Metrics: What Counts in Global Health. Durham, NC: Duke University Press, 19–54.

[bibr4-0306312719860202] Amelang K Bauer S (2019) Following the Algorithm: How epidemiological risk-scores do accountability. Social Studies of Science 49(4): 476–502. 31288646 10.1177/0306312719862049

[bibr5-0306312719860202] Armstrong D (2017) Clinical prediction and the idea of a population. Social Studies of Science 47(2): 288–299. 28105894 10.1177/0306312716685926

[bibr6-0306312719860202] Asad T (1994) Ethnographic representation, statistics and modern power. Social Research 61(1): 55–88.

[bibr7-0306312719860202] Ashmore M Mulkay M Pinch T (1989) Health and Efficiency: A Sociology of Health Economics. Bristol: Open University Press.

[bibr8-0306312719860202] Bauer S (2013) Modeling population health. Medical Anthropology Quarterly 27(4): 510–530. 24214880 10.1111/maq.12054

[bibr9-0306312719860202] Bauer S (2014) From administrative infrastructure to biomedical resource: Danish population registries, the ‘Scandinavian laboratory’, and the ‘epidemiologist’s dream’. Science in Context 27(2): 187–213. 24941789 10.1017/s0269889714000040

[bibr10-0306312719860202] Beitsch L Corso LC (2009) Accountability: The fast lane on the highway to change. American Journal of Public Health 99(9): 1545. 10.2105/AJPH.2009.172957 PMC2724452 19608935

[bibr11-0306312719860202] Bessette JM (2001) Acountability: Political. In: Smelser NJ Baltes PB (eds) International Encyclopedia of the Social & Behavioral Sciences. Amsterdam: Elsevier, 38–41.

[bibr12-0306312719860202] Bevan G Hood C (2006) What’s measured is what matters: Targets and gaming in the English public health care system. Public Administration 84(3): 517–538.

[bibr13-0306312719860202] Bigo D Isin E Ruppert E (2019) Data politics. In: Bigo D Isin E Ruppert E (eds) Data Politics: Worlds, Subjects, Rights. Abingdon: Routledge, 1–17.

[bibr14-0306312719860202] Biruk C (2018) Cooking Data: Culture and Politics in an African Researchwworld. Durham, NC: Duke University Press.

[bibr15-0306312719860202] Bowker GC Star SL (1999) Sorting Things Out: Classification and Its Consequenses. Cambridge, MA: The MIT Press.

[bibr16-0306312719860202] Brenneis D (2006) Reforming promise. In: Riles A (ed.) Documents. Ann Arbor, MI: The University of Michigan Press, 41–70.

[bibr17-0306312719860202] Brodersen J Schwartz LM Heneghan C, et al (2018) Overdiagnosis: What it is and what it isn’t? BMJ Evidence-Based Medicine 32(1): 1–3. 10.1136/ebmed-2017-110886 29367314

[bibr18-0306312719860202] Callon M Muniesa F (2005) Economic markets as calculative collective devices. Organization Studies 26(8): 1229–1250.

[bibr19-0306312719860202] Cool A (2019) Impossible, unknowable, accountable: Dramas and dilemmas of data law. Social Studies of Science 49(4): 503–530. 31057059 10.1177/0306312719846557

[bibr20-0306312719860202] Creemers R (2016) Disrupting the Chinese state: Internet plus, new actor and new factors. Asiascape: Digital Asia 5: 169–197.

[bibr21-0306312719860202] Decoteau CL Underman K (2015) Adjudicating non-knowledge in the Omnibus Autism Proceedings. Social Studies of Science 45(4): 471–500. 26502656 10.1177/0306312715600278

[bibr22-0306312719860202] Deibert RJ Pauly LW (2019) Mutual entanglement and complex sovereignty in cyberspace. In: Bigo D Isin E Ruppert E (eds) Data Politics: Worlds, Subjects, Rights. Abingdon: Routledge, 81–99.

[bibr23-0306312719860202] Deleuze G (1992) Postscript on the societies of control. October 59: 3–7.

[bibr24-0306312719860202] Denis J Goëta S (2017) Rawification and the careful generation of open government data. Social Studies of Science 47(5): 604–629. 28633611 10.1177/0306312717712473

[bibr25-0306312719860202] Desrosiéres A (1998) The Politics of Large Numbers: A History of Statistical Reasoning. Cambridge, MA: Harvard University Press.

[bibr26-0306312719860202] Desrosiéres A (2011) Words and numbers: For a sociology of the statistical argument. In: Sætnan AR Lomell HM Hammer S (eds) The Mutual Construction Statistics and Society. New York: Routledge, 41–64.

[bibr27-0306312719860202] Edwards PN Mayernik MS Batcheller AL, et al (2011) Science friction: Data, metadata, and collaboration. Social Studies of Science 41(5): 667–690. 22164720 10.1177/0306312711413314

[bibr28-0306312719860202] Erikainen S Pickersgill M Cunningham-Burley S, et al (2019) Patienthood and participation in the digital era. Digital Health 5: 2055207619845546. 31041112 10.1177/2055207619845546 PMC6482654

[bibr29-0306312719860202] Erikson SL (2012) Global health business: The production and performativity of statistics in Sierra Leone and Germany. Medical Anthropology 31(4): 367–384. 22746684 10.1080/01459740.2011.621908

[bibr30-0306312719860202] Erikson SL (2016) Metrics and market logics of global health. In: Adams V (ed.) Metrics: What Counts in Global Health. Durham, NC: Duke University Press, 147–162.

[bibr31-0306312719860202] Europa-Kommisionen (2017) Meddelelse fra kommisionen til Europa-parlamentet, rådet, det europæiske økonomiske og sociale udvalg og regionsudvalget om midtvejsevalueringen af gennemførelsen af strategien for det digitale indre marked. Et forbundet digitalt indre marked for alle. Bruxelles: Europa-Kommisionen.

[bibr32-0306312719860202] Fahlquist JN (2006) Responsibility ascriptions and public health problems. Journal of Public Health 14(1): 15–19.

[bibr33-0306312719860202] Foucault M (1991) Governmentality. In: Burchell G Gordon C Miller P (eds) The Foucault Effect: Studies in Governmentality. Chicago, IL: The University of Chicago Press, 87–104.

[bibr34-0306312719860202] Foucault M (1999) Ordene og Tingene. En Arkæologisk Undersøgelse af Videnskaberne om Mennesket [The Order of Things. An Archaeology of the Human Sciences]. Viborg: Spektrum.

[bibr35-0306312719860202] Geissler PW (2013) Public secrets in public health: Knowing not to know while making scientific knowledge. American Ethnologist 40(1): 13–34.

[bibr36-0306312719860202] Ginsburg F Rapp R (2017) Cripping the new normal: Making disability count. ALTER, European Journal of Disability Research 11(3): 179–192.

[bibr37-0306312719860202] Gitelman L Jackson V (2013) Introduction. In: Gitelman L (ed.) ‘Raw Data’ Is an Oxymoron. Cambridge, MA: The MIT Press, 1–14.

[bibr38-0306312719860202] Goëta S Davies T (2016) The daily shaping of state transparency: Standards, machine-readability and the configuration of open government data policies. Science & Technology Studies 29(4): 10–30.

[bibr39-0306312719860202] Grommé F Ruppert E (2019) Population geometries of Europe: The topologies of data cubes and grids. Science, Technology & Human Values. Epub ahead of print 10 March. DOI: 10.1177/0162243919835302.

[bibr40-0306312719860202] Hacking I (1982) Biopower and the avalanche of printed numbers. Humanities in Society 5: 279–295.

[bibr41-0306312719860202] Hacking I (1990) The Taming of Chance. Cambridge: Cambridge University Press.

[bibr42-0306312719860202] Halfon S (2006) The disunity of consensus: International population policy coordination as socio-technical practice. Social Studies of Science 36(5): 783–807.

[bibr43-0306312719860202] Henderson R Keiding N (2005) Individual survival time prediction using statistical models. Journal of Medical Ethics 31(12): 703–706. 16319233 10.1136/jme.2005.012427 PMC1734073

[bibr44-0306312719860202] Hoeyer K (2002) Conflicting notions of personhood in genetic research. Anthropology Today 18(5): 9–13. 17333599 10.1111/1467-8322.00129

[bibr45-0306312719860202] Hoeyer K (2019) Data as promise: Reconfiguring Danish public health through personalized medicine. Social Studies of Science 49(4): 531–555. 31272287 10.1177/0306312719858697

[bibr46-0306312719860202] Hogle LF (2019) Accounting for accountable care: Value-based population health management. Social Studies of Science 49(4): 556–582. 31122142 10.1177/0306312719840429

[bibr47-0306312719860202] Holmberg C Bischof C Bauer S (2013) Making predictions: Computing populations. Science, Technology, & Human Values 38(3): 398–420.

[bibr48-0306312719860202] Isin E Ruppert E (2019) Data’s empire. In: Bigo D Isin E Ruppert E (eds) Data Politics: Worlds, Subjects, Rights. Abingdon: Routledge, 207–227.

[bibr49-0306312719860202] Jaton F (2017) We get the algorithms of our ground truths: Designing referential databases in digital image processing. Social Studies of Science 47(6): 811–840. 28950802 10.1177/0306312717730428 PMC5697567

[bibr50-0306312719860202] Jerak-Zuiderent S (2015) Accountability from somewhere and for someone: Relating with care. Science as Culture 24(4): 412–435.

[bibr51-0306312719860202] Jutel A (2009) Sociology of diagnosis: A preliminary review. Sociology of Health & Illness 31(2): 278–299. 19220801 10.1111/j.1467-9566.2008.01152.x

[bibr52-0306312719860202] Kalender U Holmberg C (2019) Courtesy work: Care practices for quality assurance in a cohort study. Social Studies of Science 49(4): 583–604. 31382858 10.1177/0306312719863139

[bibr53-0306312719860202] Kant I (2017 [1781]) Critique of Pure Reason. Delhi, India: Green Bird Publications.

[bibr54-0306312719860202] Kerr A Broer T Ross E, et al (2019) Polygenic risk-stratified screening for cancer: Responsibilization in public health genomics. Social Studies of Science 49(4): 605–626. 31230567 10.1177/0306312719858404 PMC6688132

[bibr55-0306312719860202] Kraak V Swinburn B Lawrence M (2014) Distinguishing accountability from responsibility: An accountability framework. American Journal of Public Health 104(6): e2–e3. 10.2105/AJPH.2014.301899 PMC4062007 24825226

[bibr56-0306312719860202] Lambert H (2006) Accounting for EBM: Notions of evidence in medicine. Social Science & Medicine 62(11): 2633–2645. 16387399 10.1016/j.socscimed.2005.11.023

[bibr57-0306312719860202] Latour B (1999) Circulating reference: Sampling the soil in the Amazon forest. In: Latour B (ed.) Pandora’s Hope: Essays on the Reality of Science Studies. Cambridge, MA: Harvard University Press, 24–79.

[bibr58-0306312719860202] Latour B Woolgar S (1979) Laboratory Life: The Construction of Scientific Facts. Beverly Hills: Sage Publications.

[bibr59-0306312719860202] Law J (2009) Seeing like a survey. Cultural Sociology 3(2): 239–256.

[bibr60-0306312719860202] Lemke T (2007) Biopolitik zur Einführung. Hamburg: Junius Verlag.

[bibr61-0306312719860202] Lupton D (1997) The Imperative of Health: Public Health and the Regulated Body. London: SAGE.

[bibr62-0306312719860202] Lury C Day S (2019) Algorithmic personalization as a mode of individuation. Theory, Culture & Society 36(2): 17–37.

[bibr63-0306312719860202] McGoey M (2012) The logic of strategic ignorance. British Journal of Sociology 63(3): 533–576. 22950467 10.1111/j.1468-4446.2012.01424.x

[bibr64-0306312719860202] Martin A Lynch M (2009) Counting things and people: The practices and politics of counting. Social Problems 56(2): 243–266.

[bibr65-0306312719860202] Mayer-Schönberger V Cukier K (2013) Big Data: A Revolution That Will Transform How We Live, Work and Think. London: John Murray.

[bibr66-0306312719860202] Merry SE (2016) The Seductions of Quantification: Measuring Human Rights, Gender Violence, and Sex Trafficking. Chicago, IL: The University of Chicago Press.

[bibr67-0306312719860202] Moore PV (2018) The Quantified Self in Precarity Work, Technology and What Counts. New York: Routledge.

[bibr68-0306312719860202] Moreira T (2019) Devicing future populations: Problematizing the relationship between quantity and quality of life. Social Studies of Science 49(1): 118–137. 30803351 10.1177/0306312719829841

[bibr69-0306312719860202] Moreira T Palladino P (2011) ‘Population laboratories’ or ‘laboratory populations’? Making sense of the Baltimore longitudinal study of aging, 1965–1987. Studies in History and Philosophy of Biological and Biomedical Sciences 42(3): 317–327. 21802636 10.1016/j.shpsc.2011.05.001

[bibr70-0306312719860202] Muller JZ (2018) The Tyranny of Metrics. Princeton, NJ: Princeton University Press.

[bibr71-0306312719860202] Murphy M (2017) The Economization of Life. Durham, NC: Duke University Press.

[bibr72-0306312719860202] O’Riordan K (2017) Unreal Objects – Digital Materialities, Technoscientific Projects and Political Realities. London: Pluto Press.

[bibr73-0306312719860202] Olsen L Aisner D McGinnis JM (2007) The Learning Healthcare System: Workshop Summary. Washington, DC: The National Academies Press. 21452449

[bibr74-0306312719860202] Petersen A (2019) Digital Health and Technological Promise: A Sociological Inquiry. London: Routledge.

[bibr75-0306312719860202] Piantadosi S Byar DP Green SB (1988) The ecological fallacy. American Journal of Epidemiology 127(5): 893–904. 3282433 10.1093/oxfordjournals.aje.a114892

[bibr76-0306312719860202] Pickersgill M (2019) Access, accountability, and the proliferation of psychological therapy: On the introduction of the IAPT initiative and the transformation of mental healthcare. Social Studies of Science 49(4): 627–650. 30905265 10.1177/0306312719834070 PMC7613166

[bibr77-0306312719860202] Plant S (1998) Zeros + Ones. London: Fourth Estate.

[bibr78-0306312719860202] Pols J (2006) Accounting and washing: Good care in long-term psychiatry. Science, Technology & Human Values 31(4): 409–430.

[bibr79-0306312719860202] Porter TM (1995) Trust in Numbers: The Pursuit of Objectivity in Science and Public Life. Princeton, NJ: Princeton University Press. 10.1177/030631299029004007 11623934

[bibr80-0306312719860202] Power M (1997) The Audit Society: Rituals of Verification. Oxford: Oxford University Press.

[bibr81-0306312719860202] Rabeharisoa V Callon M Filipe AM, et al (2014) From ‘politics of numbers’ to ‘politics of singularisation’: Patients’ activism and engagement in research on rare diseases in France and Portugal. BioSocieties 9(2): 194–217.

[bibr82-0306312719860202] Radin J (2017) Life on Ice: A History of New Uses for Cold Blood. Chicago, IL: The University of Chicago Press.

[bibr83-0306312719860202] Reinholdt M (2018) Anticipating psychosis: The Copenhagen High-Risk Project and the dream of the prevention of schizophrenia. History of the Human Sciences 32(2): 106–217.

[bibr84-0306312719860202] Riles A (2006) Introduction. In: Riles A (ed.) Documents. Ann Arbor, MI: The University of Michigan Press, 1–38.

[bibr85-0306312719860202] Rose N (2007) The Politics of Life Itself: Biomedicine, Power, and Subjectivity in the Twenty-first Century. Princeton, NJ: Princeton University Press.

[bibr86-0306312719860202] Ruckenstein M Schüll ND (2017) The datafication of health. Annual Review of Anthropology 46: 261–278.

[bibr87-0306312719860202] Ruppert E (2012) The governmental topologies of database devices. Theory, Culture & Society 29(4–5): 116–136.

[bibr88-0306312719860202] Ruppert E Isin E Bigo D (2017) Data politics. Big Data & Society 4(2): 1–7.

[bibr89-0306312719860202] Sætnan AR Lommel HM Hammer S (2011) Introduction. By the very act of counting: The mutual construction of statistics and society. In: Sætnan AR Lommel HM Hammer S (eds) The Mutual Construction of Statistics and Society. New York: Routledge, 1–21.

[bibr90-0306312719860202] Sangaramoorthy T Benton A (2012) Enumeration, identity, and health. Medical Anthropology 31(4): 287–291. 22746679 10.1080/01459740.2011.638684

[bibr91-0306312719860202] Stark L (2018) Algorithmic psychometrics and the scalable subject. Social Studies of Science 48(2): 204–231. 29726810 10.1177/0306312718772094

[bibr92-0306312719860202] Storeng KT Behage DP (2017) ‘Guilty until proven innocent’: The contested use of maternal mortality indicators in global health. Critical Public Health 27(2): 163–176. 28392630 10.1080/09581596.2016.1259459 PMC5359740

[bibr93-0306312719860202] Strathern M (2000) Introduction: New accountabilities. In: Strathern M (ed.) Audit Cultures: Anthropological Studies in Accountability, Ethics and the Academy. London: Routledge, 1–18.

[bibr94-0306312719860202] Sullivan N (2017) Multiple accountabilities: Development cooperation, transparency, and the politics of unknowing in Tanzania’s health sector. Critical Public Health 27(2): 193–204.

[bibr95-0306312719860202] Taylor L (2017) Safety in numbers? Group privacy and big data analytics in the developing world. In: Taylor L Floridi L van der Sloot B (eds) Group Privacy. Cham: Springer, 13–36.

[bibr96-0306312719860202] Taylor L Floridi L van der Sloot B (2017) Introduction: A new perspective on privacy. In: Taylor L Floridi L van der Sloot B (eds) Group Privacy. Cham: Springer, 1–13.

[bibr97-0306312719860202] The United Nations Secretary-General’s Independent Expert Advisory Group on a Data Revolution for Sustainable Development (2014) A World That Counts. New York: United Nations.

[bibr98-0306312719860202] The White House (2013) Open Government Initiative. Washington, DC: The White House.

[bibr99-0306312719860202] Tichenor M (2016) The power of data. Global maleria governance and the Senegalese data retention strike. In: Adams V (ed.) Metrics: What Counts in Global Health. Durham, NC: Duke University Press, 105–124.

[bibr100-0306312719860202] Tupasela A Snell K Cañada JA (2015) Constructing populations in biobanking. Life Sciences, Society and Policy 11(5): 1–18. 10.1186/s40504-015-0024-0 PMC4508277 26194269

[bibr101-0306312719860202] Tutton R (2004) ‘They want to know where they came from’: Population genetics, identity, and family genealogy. New Genetics and Society 23(1): 105–120. 15470787 10.1080/1463677042000189606

[bibr102-0306312719860202] Van Belle S Mayhew SH (2016) What can we learn on public accountability from non-health disciplines: A meta-narrative review. BMJ Open 6(7): e010425. 10.1136/bmjopen-2015-010425 PMC4947774 27388347

[bibr103-0306312719860202] Wadmann S Johansen S Lind A, et al (2013) Analytical perspectives on performancebased management: An outline of theoretical assumptions in the existing literature. Health Economics, Policy and Law 8(4): 511–527. 10.1017/S174413311300011X 23506797

[bibr104-0306312719860202] Wahlberg A Rose N (2015) The governmentalization of living: Calculating global health. Economy and Society 44(1): 60–90.

[bibr105-0306312719860202] Watcher R (2017) The Digital Doctor: Hope, Hype, and Harm at the Dawn of Medicine’s Computer Age. New York: McGraw-Hill Education.

[bibr106-0306312719860202] Weber M (1947) From Max Weber: Essays in Sociology. Oxford: Oxford University Press.

[bibr107-0306312719860202] Wholey D White K Kader H (2010) Accreditation and accountability: Is the chart before the horse? American Journal of Public Health 100: 14–15. 10.2105/AJPH.2009.181123 PMC2791240 19910339

[bibr108-0306312719860202] Wyatt S (2008) Technological determinism is dead: Long live technological determinism. In: Hackett E Amsterdamska O Lynch M, et al (eds) The Handbook of Science and Technology Studies. Cambridge, MA: The MIT Press, 165–180.

[bibr109-0306312719860202] Zuboff S (2019) The Age of Surveillance Capitalism: The Fight for a Human Future at the New Frontier of Power. New York: Public Affairs.

